# FullSSR: Microsatellite Finder and Primer Designer

**DOI:** 10.1155/2016/6040124

**Published:** 2016-06-06

**Authors:** Sebastián Metz, Juan Manuel Cabrera, Eva Rueda, Federico Giri, Patricia Amavet

**Affiliations:** ^1^Laboratorio de Genética, CONICET, Facultad de Humanidades y Ciencias, Universidad Nacional del Litoral, 3000 Santa Fe, Argentina; ^2^Cátedra de Genética, Facultad de Ingeniería, Universidad Nacional de Entre Ríos, 3100 Oro Verde, Argentina; ^3^Instituto Nacional de Limnología (INALI), UNL-CONICET, 3100 Santa Fe, Argentina

## Abstract

Microsatellites are genomic sequences comprised of tandem repeats of short nucleotide motifs widely used as molecular markers in population genetics. FullSSR is a new bioinformatic tool for microsatellite (SSR) loci detection and primer design using genomic data from NGS assay. The software was tested with 2000 sequences of* Oryza sativa* shotgun sequencing project from the National Center of Biotechnology Information Trace Archive and with partial genome sequencing with ROCHE 454® from* Caiman latirostris*,* Salvator merianae*,* Aegla platensis*, and* Zilchiopsis collastinensis*. FullSSR performance was compared against other similar SSR search programs. The results of the use of this kind of approach depend on the parameters set by the user. In addition, results can be affected by the analyzed sequences because of differences among the genomes. FullSSR simplifies the detection of SSRs and primer design on a big data set. The command line interface of FullSSR was intended to be used as part of genomic analysis tools pipeline; however, it can be used as a stand-alone program because the results are easily interpreted for a nonexpert user.

## 1. Introduction

Microsatellites (also known as “Short Sequence Repeats” (SSRs)) are genomic sequences comprised of tandem repeats of short nucleotide motifs (1 to 6 bp). SSRs have been widely used as molecular markers in population biology because they have high mutation rates with high levels of polymorphism between organisms of the same population [1, 2]. Traditional methods to isolate SSRs included the construction of enriched genomic libraries, cloning, and sequencing; however this approach is time-consuming and very expensive. An alternative method is using Next Generation Sequencing (NGS) to obtain a great number of sequences reducing the costs at the same time. On each side of the SSR there are flanking regions that are critical to develop locus-specific primers to amplify the microsatellites by PCR. The design of a high number of primers is a challenge logistically both in terms of achieving good coverage of target regions and in terms of cost [3].

Nowadays there are many informatics tools to detect SSR (PHOBOS [4], MISA [5], and Tandem Repeats Finder [2]) and for specific primer design (Primer3 [6] or FastPCR [7]). In this work, we developed an informatics tool that combines SSR detection and primer design. FullSSR can detect all SSRs within a group of sequences and design primers for each SSR detected.

## 2. Materials and Methods

**Figure 1:**
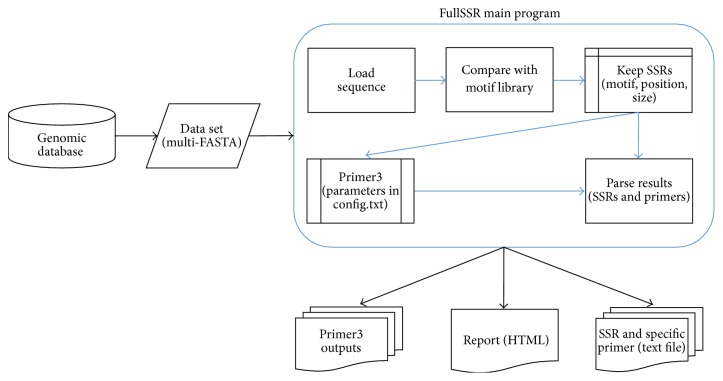
Flowchart of FullSSR. Multi-FASTA file is used as input; in the gray box are the different processing modules of the core program. There are three kinds of outputs: easy-to-interpret HTML report, Primer3 native outputs, and text files for each SSR found.

FullSSR was programmed in Perl. It consists in two main modules: SSRs search and primer design (Figure 1).

### 2.1. SSR Search

The most used algorithms for detection of microsatellites are based on three main approaches. The first approach consists in scanning genomic sequences linearly to detect tandem repeats as subsequences following several specifications [3, 8]. The second approach uses statistical rules to detect subsequences that may be microsatellites. These regions are then submitted to validation tests that sieve out desired sequences. Although this approach is time efficient, it needs appropriate statistical criteria to ensure relevant results [9, 10]. In the third approach, algorithms align a given DNA motif, or library of motifs, along genomic sequences. Regions detected as microsatellites are those whose alignment score is higher than a given threshold [11, 12].

FullSSR utilizes an algorithm that combines the first and third approach. It begins with the creation of a list of all possible combinations between two and five nucleotides (adenine, guanine, thymine, and cytosine). Then it analyzes the group of sequences searching for all the elements of the motif list. All the matches are filtered using predefined rules (e.g., in case of two overlapped SSRs it will only report the longer one).

The results show the ID of the analyzed sequence, the repeated sequence (motif), the number of tandem repeats, the length of the SSR, and the position of it within the sequence (start, end). Imperfect microsatellites are generally excluded from studies; this provide a biased view of reality, because imperfections result from the evolutionary process and influence the evolutionary dynamics by restricting the slippage rate [13, 14].

Previous experience at lab-work has shown that imperfect microsatellites are nonuseful because they are difficult to optimize and not easily amplified by PCR. FullSSR discards imperfect microsatellites.

### 2.2. Primer Design

For primer design FullSSR uses a BioPerl package modification [15] (Bio::Tools::Run::Primer3) in order to create an interface to run Primer3 using previously detected SSRs. Primer3 native output is stored and used to create an easy-to-read report where primers are shown alongside the respective SSR. The parameters that Primer3 uses for the oligonucleotides design, such as melting temperature (*T*_*m*_), optimum GC content, and product and primer size, can be modified with a configuration file.

## 3. Results and Discussion

### 3.1. Execution

This tool runs under UNIX command line interface. It requires the installation of Perl, BioPerl, and Primer3.

### 3.2. Data Entry

This software was tested with 2000 sequences of* Oryza sativa* shotgun sequencing projects from the NCBI (National Center of Biotechnology Information) Trace Archive [16]. The results were compared with other SSR search tools. We also perform an analysis on partial genome sequencing with ROCHE 454 from reptiles as* Caiman latirostris* and* Salvator merianae* and freshwater crabs* Aegla platensis* and* Zilchiopsis collastinensis*, in order to evaluate the software with different kinds of taxa. All of them are species that inhabit Argentine wetlands, with unknown genomes. We purified DNA from muscle tissue (500 ng/*μ*L final concentration) of the four species.

### 3.3. Microsatellite Search

A total of 908 microsatellites were found in 2000* O. sativa* sequences. The software filtrate repeated very short and imperfect SSRs. We compare these results with MISA and PHOBOS results using similar configuration (Table 1).

Even though the amount of microsatellites found by MISA and PHOBOS is much higher than the amount found by the search algorithm used in FullSSR, most of them are not suitable for primer design, since they are located near the beginning or the end of the sequence, making it impossible to design primers for both sides of the microsatellite.

Data, from partial genome sequencing of reptiles and crabs, shows that results may vary depending the sequence under consideration because of differences among the genomes, for example, structure, GC content, and gene composition (Tables 2 and 3).

### 3.4. Primer Design

Primer design involved default parameters of Primer3. From 428 SSRs of* O. sativa*, 343 primer pairs were designed. The primers are shown in an HTML report with global statistics and the primer sequence and its characteristics (*T*_*m*_, % GC, stability, product size, etc.).

Primers obtained from 8 SSRs of* Caiman latirostris* sequences were wet-lab tested and proved to be suitable for molecular genetic studies. The results were reported in a previous work [17].

## 4. Conclusions

**Figure 2:**
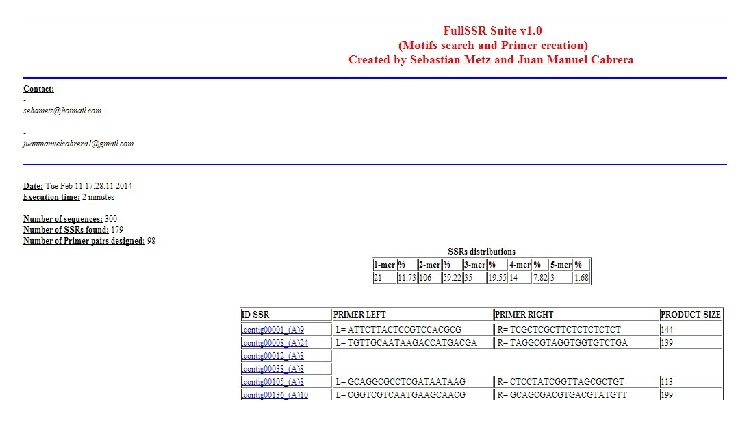
Screenshot of the HTML report of the program. SSR distribution and primers for each SSR are shown (if any). Additional data is displayed (execution time, number of sequences, etc.).

FullSSR simplifies the detection of SSRs and primer design on a big data set. The command line interface of FullSSR was intended to be used as part of genomic analysis tools pipeline (command line interfaces are generally easier to automate via scripting); however, it can be used as a stand-alone program because the results are easily interpreted for nonexpert user (Figure 2). Other tools as Msatcommander [18] and QDD [19] have a user interface that makes them not available for automated use. Pal finder [20] shares functions with FullSSR but it uses an old version of Primer3.

## 5. Availability and Requirements

- Project name: FullSSR.
- Project home page: https://sourceforge.net/projects/fullssr/.
- Operating system(s): Linux OS.
- Programming language: Perl.
- Other requirements: BioPerl and Primer3 ver. 2.3.7.
- License: GNU General Public License (GNU-GPL).
- Any restrictions to use by nonacademics: none.

## 6. Availability of Supporting Data

We obtained* O. sativa* data set supporting the results of this paper performing a query in the National Center of Biotechnology Information (NCBI) Trace Archive database http://www.ncbi.nlm.nih.gov/Traces/trace.cgi. The submitted query was SPECIES CODE = “ORYZA SATIVA (INDICA CULTIVAR-GROUP)” AND TRACE TYPE CODE = “SHOTGUN” and we retrieve the first 2000 sequences.

The sequences of* Caiman latirostris* primers are included in GenBank (Accession numbers: KP849485–KP849492) and have been recently published [17].

## Figures and Tables

**Table 1:** Algorithm comparison: results comparison among FullSSR, MISA, and PHOBOS using *O. sativa* sequences.

*O. sativa*	FullSSR	MISA	PHOBOS
1-mer	615	812	1518
2-mer	172	831	832
3-mer	101	275	1435
4-mer	17	31	774
5-mer	3	7	828
Total	908	1956	5387

**Table 2:** SSR classification. SSR types obtained with sequences from different partial sequencing projects. The 1-mer SSRs found are greater than 8 bp long.

*O. sativa*	*Aegla platensis*	*Zilchiopsis collastinensis*	*Caiman latirostris*	*Salvator merianae*
1-mer	21	175	33	56
2-mer	106	644	77	139
3-mer	35	146	14	33
4-mer	14	30	4	25
5-mer	3	5	4	6
Total	179	1000	131	5387

**Table 3:** Results for different data set. bp read: base pairs read; % GC: guanine-cytosine percentage; SSRs and primers: number of SSRs found and number of primers designed.

Species	bp read	% GC	SSRs	Primers
*Aegla platensis*	143637	41,34	179	98
*Zilchiopsis collastinensis*	907553	42,12	1000	605
*Caiman latirostris*	308323	44,65	131	92
*Salvator merianae*	275101	50,23	259	148

## Abbreviations

SSRs: Short Sequence Repeats
PCR: Polymerase Chain Reaction
NGS: Next Generation Sequencing.
